# The effect of health-care Qigong Baduanjin combined with auricular point sticking on athletes’ pre-competition anxiety: A protocol for systematic review and meta-analysis

**DOI:** 10.1097/MD.0000000000024874

**Published:** 2021-02-19

**Authors:** Lujia Li, Xiaozheng Li, Pingping Xie, Yan Li, Li Ma, Baoyu Ding

**Affiliations:** a School of Sport Training Science, Tianjin University of Sport, No. 16 Donghai Road, West District of Tuanbo New Town, Jinghai District; b Department of Psychiatric, Tianjin Anding Hospital, No. 13 Liulin Road, Hexi District; c School of Sports and Culture, Tianjin University of Sport, No. 16 Donghai Road, West District of Tuanbo New Town, Jinghai District; d Department of Internal Medicine of Traditional Chinese Medicine; e Department of Rehabilitation, Tianjin Binhai New Area of Hangu Hospital of Traditional Chinese Medicine, No. 38 Hangupaifang East Street, Binhai New Area, Tianjin, China.

**Keywords:** athletes, auricular point sticking, health-care Qigong Baduanjin, meta-analysis, pre-competition anxiety, pre-competition state anxiety

## Abstract

**Background:**

Competition anxiety is also known as pre-competition anxiety (PCA), because this anxiety state often occurs before the athletes face the competition. If it is not adjusted in time, which will greatly affect the performance of athletes, even the mental health and physical health of athletes. Therefore, the selection of appropriate methods to intervene the athletes, reducing the PCA of athletes, and it has an important effect on the competition performance of athletes. Therefore, based on the basic theory of traditional Chinese medicine and sports psychology principles, this study adopts a way of systematic evaluation to study the effect of health-care Qigong Baduanjin (HCQB) combined with auricular point sticking (APS)in the treatment of athletes’ PCA (APA), the purpose is to help the majority of athletes to eliminate the PCA.

**Methods:**

Two searchers independently retrieve CNKI, WANFANG databases, VIP, CBM, Web of Science, Embase, PubMed, The Cochran Library and other Chinese and English databases. It is supplemented by manual retrieval to comprehensively collect the relevant literature data of the clinical controlled study of HCQB combined with APS in the treatment of APA. The retrieval time is from January 1, 1990 to October 1, 2020, using the subject word and keywords to retrieve, developing a retrieval style according to the characteristics of the database. The two evaluators independently use the above-mentioned retrieval methods to retrieve the main literature database, summarizing and removing the duplicate literature, then reading the title and abstract of the literature separately, excluding the literature that clearly does not meet the inclusion criteria, and finally reading the literature, and finally including the literature in line with the study, in case of disagreement, with the third researcher to decide. The quality evaluation of the literature is independently evaluated using the bias risk assessment criteria for randomized controlled trials in Cochrane Manual 5.1.0. Using the RevMan 5.3 software for meta-analysis.

**Results:**

This study will study the effect of HCQB combined with APS on reducing APA, and the results of the study will be published in high-impact academic journals.

**Conclusion:**

The quality of athletes’ mental state is related to whether athletes can play their true level of sports in the competition, and good mental state is also the prerequisite to ensure that athletes get better results. The conclusions reached by this study will provide quantifiable reference for coaches and athletes, with the aim of providing theoretical basis for helping the athletes eliminate PCA.

**Ethics and dissemination:**

The type of this study belongs to the category of systematic evaluation, the data in this study are derived from published research papers and public data in the Internet, so ethical review is not suitable for this study.

**PROSPERO registration number:**

2021 CRD42021228254.

## 1 Introduction

Anxiety is a state of nervousness and fear that causes self-esteem and self-confidence to be frustrated or increases feelings of failure and guilt due to the inability to reach goals or overcome the threat of obstacles. Psychologically manifested as an emotional state of restlessness, anxiety, tension, and fear.^[1,2]^ Physiologically, it is accompanied by an increase in arousal levels, characterized by different intensity and constant changes over time, and different anxiety is generated by motivation under different conditions. Pre-examination anxiety and pre-competition anxiety (PCA), are a common psychological phenomenon. In today's sports world, the psychological training of outstanding athletes has received more and more international attention. In various competitive sports, while emphasizing the improvement of scientific research level and skills, the psychological training of athletes will also be the focus of discussion.^[3,4]^ Mr. Gruber of the United States pointed out at the Olympic Science Conference that “the psychological factors of low-level and middle-level athletes account for 20% of skills and 80% of biometric factors, while the psychological factors of good athletes account for 80% and biometric factors account for 20%.” In this way, mental state plays an important role in success or failure of high-level competitions.^[5]^

Sports anxiety is a tendency to worry about current or anticipated situational manifestations of potential threats to self-esteem in training and competition, manifested as emotional states such as restlessness, anxiety, tension, and fear, which are physically accompanied by an increase in arousal levels. Its main features are different intensity and constant changes over time. The uncertainty of sports competition is the root cause of sports anxiety.^[6,7]^ Sports anxiety is associated with arousal levels and tension state. In sports and competitions, the first step is to maintain a certain state of arousal or awakening, so that it reaches a suitable level of anxiety. If there is no certain level of awakening or tension, psychological activity will not have a certain direction, the cerebral cortex will not easily form a dominant excitement center and increase the degree of concentration, the function of the body's motor organs and intellectual factors cannot be in the best working state, showing low sensitivity for muscle discrimination, weakened observation ability, slow thinking, slow reaction, and large movement errors and other symptoms. Therefore, the low level of anxiety is not conducive to sports competition.^[8]^ However, high levels of anxiety can interfere with attention, disrupt temporary memory, and produce negative awareness. Players with high anxiety tend to pay too much attention to the possibility of failure of action exercises or the results of competitions, thus blocking cognitive processing activities, resulting in a decrease in the efficiency of information processing, but also affecting the learning of complex motion skills or sports performance.^[9]^ Therefore, choosing the right method to intervene in the anxiety state of athletes before the game has also become an important link to determine athletes’ performance, and it is also one of the hot topics in sports medicine study.

Health-care qigong is China's national traditional health sports, which is an important part of the Chinese nation's long history and culture, including: Baduanjin, Yijinjing, Five-Animal Exercises and Liuzijue. Its exercise methods are deeply loved by the general public. Contemporary meaning for health-care qigong emphasizes physical exercise as a method of exercise aimed at improving physical and mental health and improving physical function.^[10]^ Health-care qigong in the concept of fitness and fitness methods in line with the traditional Chinese medicine stressed the concept of “the unity of body and spirit,” “harmony of spirt and mind” and “preventive disease.” Psychologically, it can adjust to improve people's bad psychological state, physiologically, it can enhance the function of human organs, improve body function, and enhance the ability of disease prevention and anti-aging.^[11]^ Qigong Baduanjin is an independent and complete traditional health-care qigong method in China, which originated in the Northern Song Dynasty, and it has a total of 800 years of history, and has a good effect on strengthening physical fitness and improve the psychological state. This method is divided into 8 paragraphs, each paragraph is an action, so called “Baduanjin,” at the same time, Baduanjin is one of the treasures of medical sports in China. It belongs to aerobic exercise with less exercise intensity, mainly guiding and stretching. But it is not just pure sports, but closely combining and organic using of physical fitness, breathing, mind of the three exercise methods at the same time to work together. In the exercise of Baduanjin, the individual can quickly enter a stable and rhythmic breathing state, and the breathing gradually becomes gentle, deep and even, and the respiratory rate gradually slows down, so that the individual can reach a relaxed state and relieve and eliminate anxiety and other adverse emotions. In the exercise of Baduanjin, it also highlights the emotional adjustment, which requires calm emotions, a quiet and tranquil state, and focuses attention on one's own body, and it emphasizes the combination of mind and spirit, moves along the meridians, and postures along the meridians, so as to make the brain gradually reach a specific mental activity, which is tight and orderly. It requires the relaxation of the body's muscles, mental will, and mental inwardness, to achieve a relaxed state.^[12,13]^ Auricular point sticking (APS) is also known as auricular point compression therapy, to determine the main auxiliary point according to the condition, and adheres to the acupoints with adhesive tape, such as saponaria vaccaria, magnetic beads, etc., to give a moderate kneading, pressing, pinching, pressure, so that it produces heat, hemp, swelling, pain and other stimulation, in order to achieve the treatment purpose of external treatment.^[14]^

At present, health-care Qigong Baduanjin (HCQB) and APS have been applied in the treatment of athletes’ PCA (APA), but there is some controversy about its effectiveness, but also due to the lack of high-quality experimental study. Therefore, the widespread use of these technologies is still limited. This study uses the method of systematic evaluation to study the effects of HCQB combined with APS in the treatment of APA. The purpose of this study is to find suitable and effective ways to relieve APA, so that more athletes can get rid of the effects of PCA and achieve better results in the competition.

## 2 Methods

### 2.1 Protocol registration

PROSPERO registration number: 2021 CRD42021228254. Available from: https://www.crd.york.ac.uk/prospero/display_record.php?ID=CRD42021228254. This study was carried out in full accordance with the requirements of the Preferred Reporting Items for Systematic Review and Meta-Analysis Protocols checklist.

### 2.2 Inclusion and exclusion criteria

#### 2.2.1 Types of studies

A randomized controlled study on the effects of HCQB combined with APS on APA is collected, and the literature is retrieved as a Chinese and English database, and the published language of the literature is set to Chinese or English.

Randomized controlled trials (RCTs) are selected according to the manual examination guidelines developed by the international Cochrane Collaboration Network, and relevant RCT articles meeting the requirements are retrieved:

(1) A study is conducted in one or more patients;
(2) Two or more interventions are compared over the same period;
(3) RCT: clinical trials in which subjects are assigned to different treatment groups using randomized allocation methods (random number tables, computer randomization, drawing lots, coin tosses, etc).

#### 2.2.2 Types of participant

Athletes who will participate in the competition in the short term, combined with the concept of PCA and clinical performance, the diagnosis of PCA is mainly based on the diagnosis criteria of general anxiety disorder in the “Diagnostic and Statistical Manual of Mental Disorders (Fifth Edition).” the age of the patient, the training years, the number of competitions, the type of sports competition are not limited.

#### 2.2.3 Inclusion criteria

1. It conforms to the criteria for the diagnosis of a wide range of anxiety disorders in Diagnostic and Statistical Manual of Mental Disorders (Fifth Edition):
  (1) In the past or in the past period of time, they have shown anxiety, worry, fear (anxiety expectations) for upcoming sports competitions that they will participate in.
  (2) Individuals cannot fully control this anxiety, worry, and fear.
  (3) This emotion is associated with at least 3 of the following symptoms: (1) Excited, nervous, irritable, irritable about the upcoming game. (2) Rapid heartbeat, chest tightness, shortness of breath, throat obstruction, cold hands, frequent urination; poor sleep, difficult to fall asleep, dreamy, easy to wake up. (3) Changes in appetite, upper abdominal discomfort, nausea, diarrhea and other gastrointestinal reactions. (4) Easy tiredness, difficulty concentrating or even brain blanks, or dizziness, headache. (5) Muscles cannot relax, tension stiffness, poor coordination.
  (4) This anxiety, worry, fear, or physical symptoms cause clinically significant pain or may lead to disorders in sports competitions.
  (5) This disorder cannot be attributed to the physiological effects of a substance or other physical diseases.
  (6) This disorder cannot be better explained by other mental disorders.
2. Never before have received the treatment of HCQB or APS.
3. Participating voluntarily in the study and signing an informed consent.

#### 2.2.4 Exclusion criteria

1. There is a clear physical illness.
2. People with mental illness, self-rating depression scale ≥50 points, or they are currently receiving other interventions due to PCA.
3. Those who have recently experienced major life events that may affect their emotions.
4. Patients with severe skin disease, ear skin damage, hypersensitivity to adhesive tape or saponaria vaccaria seed.
5. To ensure the reliability of the study results, the self-rating depression scale is used in this study to exclude athletes with depressive tendencies.

#### 2.2.5 Control interventions

The intervention is APS, acuity selection and the time of APS, the overall intervention time is not limited.

#### 2.2.6 Experimental interventions

**Figure 1:**
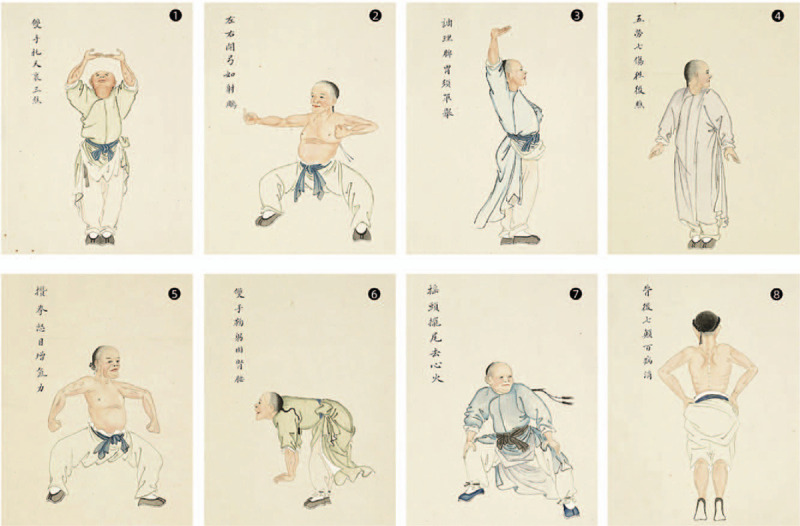
Simple flowchart of health-care Qigong Baduanjin.

The intervention is HCQB combined with APS. The requirements are the same as those in the control group. The exercise sketch of HCQB can be seen in Figure 1.

### 2.3 Types of outcome measures

#### 2.3.1 Primary outcomes

Competition state anxiety score: this experiment adopts the revised version of the competitive state anxiety inventory-2 to evaluate the athletes’ anxiety state before the competition by means of the questionnaire.^[15]^ The scale is composed of three subscales, each of which has 9 items. The score is between 9 and 36. The higher the score is, the higher the cognitive state anxiety, physical state anxiety and state self-confidence are. It is carried out by self-report method, which can be used for individual measurement and group measurement. The subscales contain three main items: cognitive state anxiety, physical state anxiety and state self-confidence. Reverse questions need to be scored in reverse direction. The answers to “not at all” is scored as 1 point, “slight” is scored as 2 points, “strong” is scored as 3 points, and “very strong” is scored as 4 points. If one question in each subscale is not answered, the average score of the 8 questions answered can be calculated firstly, and then the average number is multiplied by 9 to get the integer (if two or more questions are not answered, the questionnaire is invalid).

#### 2.3.2 Additional outcomes

Blood pressure and heart rate (measured and recorded by subjects 1, 2 and 3 weeks prior to the start of the study respectively on the day of the competition). Each time blood pressure and heart rate data are collected twice, the average value is taken).

### 2.4 Search strategy

This study follows the Preferred Reporting Items for Systematic Review and Meta-Analysis statement, which systematically retrieves Chinese and English databases such as CNKI, WANFANG databases, VIP, CBM, Web of Science, Embase, PubMed, The Cochran Library. It is supplemented by manual retrieval to comprehensively collect the relevant literature data of the clinical controlled study of HCQB combined with APS in the treatment of APA. The English retrieval words are “health-care Qigong,” “Baduanjin,” “auricular point sticking,” “auriculotherapy,” the Chinese retrieval words are “Jian-shen-qi-gong,” “Er-xue-tie-ya,” “Yun-dong-yuan-sai-qian-jiao-lv” and so on. The retrieval time is from 1 January 1990 to 1 October 2020. Taking the Cochran library database as an example to show the complete literature retrieval process, the retrieval results are shown in Table 1.

**Table 1:** Search strategy for the Cochrane Library.

Number	Search strategy
#1	(“Qigong”):ti,ab,kw OR (health Qigong):ti,ab,kw OR (fitness Qigong):ti,ab,kw OR (health-care Qigong):ti,ab,k OR (gymnastic Qigong):ti,ab,kw OR (Qi Gong):ti,ab,kw OR (Ch’i Kung):ti,ab,kw
#2	(Baduanjin):ti,ab,kw
#3	#1 AND #2
#4	(auricular point sticking):ti,ab,kw OR (auriculotherapies):ti,ab,kw OR (auriculotherapy):ti,ab,kw
#5	(athletes):ti,ab,kw OR (athlete):ti,ab,kw (professional athletes):ti,ab,kw OR (athlete, professional):ti,ab,kw OR (athletes, professional):ti,ab,kw OR (professional athlete):ti,ab,kw OR (elite athletes):ti,ab,kw OR (athlete, elite):ti,ab,kw OR (athletes, elite):ti,ab,kw OR (elite athlete):ti,ab,kw
#6	(pre-competition anxiety):ti,ab,kw OR (sports anxiety disorder):ti,ab,kw OR (sports anxiety):ti,ab,kw
#7	#5 AND #6
#8	#3 AND #4 AND #7

### 2.5 Data collection and analysis

**Figure 2:**
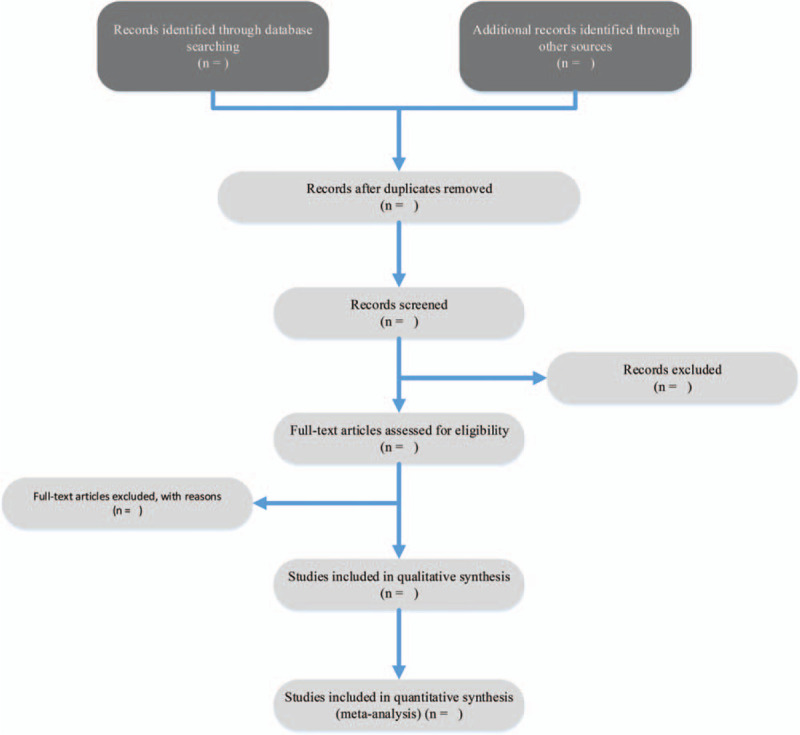
Flow chart of literature screening process.

In strict accordance with the established inclusion criteria and exclusion criteria, two researchers conduct a separate literature retrieving and screening. The literature screening and inclusion step (Fig. 2):

(1) Retrieving and downloading the full text of literature in the database according to the retrieval word.
(2) Initial screening: the literature retrieved from different databases is imported into Note Express 3.0 document management software, excluding duplicate literature; the titles of literatures are read, and the literatures such as reviews, personal experience summary and animal experiments are deleted.
(3) Secondary screening: reading the literature summary, the full text, according to the inclusion and exclusion criteria, screening the literature. In the event of a difference of opinion, the decision will be made by the third researcher.
(4) The remaining literature will be established in an Excel database, including topics, authors, year of publication, magazines, sample size, interventions, courses of treatment, observation indicators, and so on.

### 2.6 Risk of bias assessment

The studies included in this meta-analysis are RCT that evaluate the quality of literature using the evaluation criteria in the Cochrane manual, including the following six items: A. sequence generation, B. allocating concealment, sequence generation and allocating concealment are to prevent the generation of selection bias, which is caused by the different selection criteria, and it may lead to the role of intervention is overestimation or underestimation; C. blinding refers to the fact that in order to prevent the generation of implementation bias and measurement bias, the study subjects and researchers do not understand the grouping of trials, and the study designer arranges and controls all trials. D. incomplete outcome data, incomplete result data will lead to follow-up bias, which refers to the elimination of data due to patient withdrawal or loss during follow-up during or later in the trial, and the relevant data and reasons not stated in the literature or stated but not analyzed when included in the study; E. no selective outcome reporting will lead to reporting bias, due to the results reported in the article have selective reporting results; F. other sources of bias refer to other issues that may result in a high bias risk. Each evaluation entry has a “High”, “Unclear” and “Low” 3 levels of bias risk. “High” means no implementation of allocating concealment grouping, interventions are not uniform, subjects withdraw from the trial study, random method errors, inconsistent methods of measurement results, no blind method, incomplete outcome data, or not reporting the main key outcomes that should be included; “Unclear” means that the specific situation or incomplete information is not stated in the text, “Low” means that the random method describes it in detail and correctly, the use of appropriate blind method, or whether blind method is used or not will not affect the experiment results, the outcome data is complete, or the missing data does not affect the outcome analysis, and there are no other problems.

### 2.7 Statistical analysis

The Review Manager 5.3 software provided by Cochrane Collaboration Network is used to conduct meta-analysis on the final included literatures.

#### 2.7.1 Consolidation of statistics

The study data included in the literature are divided into hierarchical data and continuous variable data, and the method of statistical data is different according to the data type.

(1) When the study data included in the literature are hierarchical data, it is necessary to convert the hierarchical data into the binary variable data, and then selecting the more appropriate combined effects in OR, AR or RR for statistical analysis. OR is usually used in case-controlled studies and RR is suitable for cohort studies or randomized controlled trials.
(2) When the study data included in the literature are continuous variable data, if the units of the data are consistent, then the combined effect of MD is used for statistical analysis, and if the units of the data are different, the combined effect of SMD is used for statistical analysis.

#### 2.7.2 Dealing with missing data

If the included literature only publishes the research results without specific data information or the data published in the literature is incomplete, we will contact the corresponding author of the literature by email to obtain the original data. If the corresponding author of the paper cannot be contacted, we will only conduct statistical processing on the available data in the literature, and analyze the impact of missing data in the literature on the overall results.

#### 2.7.3 Heterogeneity Test

According to the statistical principles of meta-analysis, there is heterogeneity between the data in different studies, and we need to conduct the heterogeneity test of the included data. When there is good homogeneity between the data of multiple studies, the result indicators of heterogeneity test should show that the P value is greater than 0.10, I^2^ is less than 50%, indicating that the heterogeneity between multiple data is within the allowable range, when combining the data, fixed effect model should be chosen. If the heterogeneity between the data in multiple studies exceeds the allowable range, that is, the result indicators of the test shows that *P* ≤.10, *I*^2^≥50%, and the source of heterogeneity can be found through subgroup analysis, or carrying out sensitivity analysis to determine whether the results are accurate. If subgroup analysis or sensitivity analysis is performed, the test result indicators have changed significantly compared with the previous ones and have entered the allowable range, then it can continue to choose a fixed effect model for the data consolidation, on the other hand, if the heterogeneity test results indicators have not changed significantly and are still outside the allowable range, it should be selected with random effect model for the data consolidation. Because the random effect model analysis has the characteristic of quantifying the bias between studies, it can remove the heterogeneity caused by bias and eliminate the influence of heterogeneity between the data of different studies on the overall effect amount, so that the estimate of the effect amount is closer to the true value.

#### 2.7.4 Sensitivity analysis

Sensitivity analysis of meta-analysis is an important way to assess the reliability of results and to assess whether the combined results are affected by a single study. The methods of sensitivity analysis include: changing the analysis model, excluding literature article by article, or using other statistical methods. One by one excluding each included study, or excluding a low-quality study and then combining the effects, if there is no significant change from the previous results, the results of meta-analysis are reliable; on the contrary, if the difference is large, it indicates high sensitivity, the results of this meta-analysis are less reliable, and it needs to carefully analyze the experimental design, sample size, outcome indicators, and so on.

#### 2.7.5 Subgroup analysis

This study will be based on the patient's age, gender, have or not competition experience, athletes specifically engaged in sports, the training time and frequency of HCQB, APS in the selection of points and the outcome indicators have a clear correlation with the factors as a group basis for the development of subgroup analysis.

### 2.8 Publication Bias

This study evaluates the publication bias of the outcome indicator by making a funnel plot by Review Manager 5.3, which uses the therapy effect as a horizontal coordinate, the sample size as a ordinate, and testing the publication bias by observing the symmetry of its scatter plot. When most of the scatter points in the funnel plot are at the top or center and symmetrical to the left and right, indicating that the publication bias is not obvious, whereas the publication bias is obvious. The number of literatures is less than 7, which is not recommended as a funnel plot.

### 2.9 Assessment of the quality of evidence

The GRADE 3.2 system is used to rate the quality of evidence for the literature outcome indicators. The five downgrading conditions of the study are mainly considered: limitations, inconsistency of research results, indirectness of evidence, inaccuracy of research results, and publication bias. Three upgrade conditions: effect size, dose-effect relationship, and all confounding relationships to assess the level of evidence quality. The quality of evidence is divided into the following four levels: high quality, medium quality, low quality, and very low quality.

## 3 Discussion

The effect of anxiety on athletic performance has long been recognized by sports psychologists and coaches, and people have made unremitting efforts in measurement, evaluation and control. According to some clinical psychologists, anxiety is a negative emotion that has a negative effect on behavior. There are many models of competition anxiety, and most scholars believe that the effect of anxiety state on operational performance is complex. Each person has different levels of anxiety about the game, and different control over anxiety, resulting that anxiety state has different effects on the skill level of the athletes in the competition. However, the appropriate level of anxiety can have a positive effect on operational performance, the perception and control of anxiety is also very important, which can adjust the anxiety state under high pressure, playing a good technical level to complete the game, and that is an excellent athlete. Sports competition is a highly competitive and highly confrontational competitive sport, before and during the competition, athletes are under great psychological pressure, and it is easy to induce anxiety in a high state of tension. High level of anxiety often makes athletes’ psychological activities reach the level of extreme tension, producing premature or excessive excitement. PCA state is a state with bad emotions and has negative impact on athletic performance, it is not only a psychological experience, the body will also show different symptoms, such as dizziness, reflecting retardation, body unconscious tremors, urine frequency and other symptoms, these problems in most athletes have more or less performance, everyone's reaction is different. PCA state is most obviously manifested in two aspects, on the one hand, excessive anxiety makes the body has been in a physiologically active state, constantly consuming physical strength, so that the competition state is not good. On the other hand, the state of PCA before the game will make the athletes flustered after entering the game, the problems that arise cannot be calmly judged to deal with, high anxiety situations will also affect the ability to think.^[16]^ At present, the research on the causes, factors and effective interventions of sports anxiety at home and abroad shows a diversified trend.

HCQB and APS in China has a long history, and they are based on the traditional theory of Chinese medicine, and are widely used in the treatment of related disease, and achieved good results. In the process of practice, they have been improved and expanded. In recent years, they have been used to reduce the anxiety of athletes before competition. The long-term training of HCQB can enhance the ability of self-control, relieve brain fatigue, regulate mood, maintain physical and mental balance, the effect on mood has a positive effect. The possible mechanism is that exercise can improve the biological current and body activity of the human body, improve nervous system function, make the brain waves in various areas of the brain tend to be synchronized, the electromagnetic activity of brain cells is highly ordered, and the nerve conduction is accelerated.^[17]^ There are no relevant literatures at home and abroad devoted to the treatment of anxiety in auricular point, more reports are based on the results of the experiment to speculate on its rationale. The current research basically believes that the anti-anxiety effect of auricular points may be related to the excitement of small nerve fibers, resulting in impulse transmission to the spinal cord, midbrain, pituitary gland and hypothalamus.^[18,19]^ The certain endorphins are then released into the blood. Many neurotransmitters such as serotonin, norepinephrine, γ-aminobutyric acid, etc. will be released, and interfere with the transmission of stress signals to the central nervous system, thereby playing an anti-anxiety and anti-stress effect. However, there are still differences in the evaluation of the practical effect of these two interventions at this stage. Therefore, this study uses meta-analysis method to study the application effect of HCQB combined with APS on reducing APA, and the aim is to provide theoretical basis for finding suitable and effective methods to alleviate APA.

## Author contributions

**Conceptualization:** Lujia Li, Baoyu Ding.

**Data curation:** Lujia Li, Xiaozheng Li.

**Formal analysis:** Lujia Li, Xiaozheng Li.

**Funding acquisition:** Baoyu Ding.

**Resources:** Lujia Li, Xiaozheng Li, Pingping Xie, Yan Li, Li Ma.

**Software:** Lujia Li, Xiaozheng Li, Pingping Xie, Yan Li, Li Ma.

**Supervision:** Lujia Li.

**Writing – original draft:** Lujia Li, Xiaozheng Li, Pingping Xie, Yan Li, Li Ma.

**Writing – review & editing:** Baoyu Ding.
